# Zipf–Mandelbrot law, *f*-divergences and the Jensen-type interpolating inequalities

**DOI:** 10.1186/s13660-018-1625-y

**Published:** 2018-02-08

**Authors:** Neda Lovričević, Ðilda Pečarić, Josip Pečarić

**Affiliations:** 1 Faculty of Civil Engineering, Architecture and Geodesy, University of Split, Split, Croatia; 2 Catholic University of Croatia, Zagreb, Croatia; 3 Faculty of Textile Technology, University of Zagreb, Zagreb, Croatia; 4 RUDN University, Moscow, Russia

**Keywords:** 94A15, 94A17, 26D15, 26A51, Jensen inequality, Zipf and Zipf–Mandelbrot law, Csiszár divergence functional, *f*-divergences, Kullback–Leibler divergence

## Abstract

Motivated by the method of interpolating inequalities that makes use of the improved Jensen-type inequalities, in this paper we integrate this approach with the well known Zipf–Mandelbrot law applied to various types of *f*-divergences and distances, such are Kullback–Leibler divergence, Hellinger distance, Bhattacharyya distance (via coefficient), $\chi^{2}$-divergence, total variation distance and triangular discrimination. Addressing these applications, we firstly deduce general results of the type for the Csiszár divergence functional from which the listed divergences originate. When presenting the analyzed inequalities for the Zipf–Mandelbrot law, we accentuate its special form, the Zipf law with its specific role in linguistics. We introduce this aspect through the Zipfian word distribution associated to the English and Russian languages, using the obtained bounds for the Kullback–Leibler divergence.

## Introduction

Let us start with the notion of *f*-divergences which measure the distance between two probability distributions by making an average value, which is weighted by a specific function, of the odds ratio given by two probability distributions. Among the existing *f*-divergences introduced in the process of finding the adequate distance between two probability distributions, let us point out the Csiszár *f*-divergence [1, 2], some special cases of which are the Kullback–Leibler divergence (see [3, 4]), the Hellinger distance, the Bhattacharyya distance, the total variation distance, the triangular discrimination (see [5, 6]). The notion of ‘distance’ mainly appears as somewhat stronger than ‘divergence’ since it suggests the properties of symmetry and the triangle inequality. Considering a great number of fields in which probability theory cooperates, it is no wonder that divergences between probability distributions have many specific applications in a variety of those fields.

Jensen’s inequality, on the other hand, with its numerous refinements, variants and improvements is often called ‘a king inequality’, obviously not without a reason. Here we try to integrate one of such results concerning Jensen’s inequality in order to obtain new estimates for mentioned divergences (which deal with the convex functions for the most part). It is well known that the Jensen inequality

1 $$ f \Biggl(\frac{1}{P_{n}}\sum_{i=1}^{n}p_{i}x_{i} \Biggr)\leq\frac {1}{P_{n}}\sum_{i=1}^{n}p_{i}f(x_{i}) $$

holds for a convex function $f \colon I\rightarrow\mathbb{R}$, $I\subseteq\mathbb{R}$, an *n*-tuple $\mathbf{x}=(x_{1},\ldots ,x_{n})\in I^{n}$, $n\geq2$ and nonnegative *n*-tuple $\mathbf {p}=(p_{1},\ldots,p_{n})$, such that $P_{n}=\sum_{i=1}^{n}p_{i}> 0$.

Here we cite a result of Pečarić [7, p. 717] who investigated the method of interpolating inequalities which have reverse inequalities of Aczél type. Using Jensen’s inequality and its reverse he proved the main and the following deduced result, which holds for a convex function *f* defined on an interval $I\subset\mathbb{R}$:

2 $$\begin{aligned} & \min_{1\leq i\leq n}\{p_{i}\} \Biggl[ \sum _{i=1}^{n}f(x_{i})-nf \Biggl( \frac{1}{n}\sum_{i=1}^{n}x_{i} \Biggr) \Biggr] \\ &\quad \leq\sum_{i=1}^{n}p_{i}f(x_{i})-P_{n}f \Biggl(\frac {1}{P_{n}}\sum_{i=1}^{n}p_{i}x_{i} \Biggr) \\ &\quad\leq\max_{1\leq i\leq n}\{p_{i}\} \Biggl[ \sum_{i=1}^{n}f(x_{i})-nf \Biggl( \frac{1}{n}\sum_{i=1}^{n}x_{i} \Biggr) \Biggr], \end{aligned}$$

where $\mathbf{x}=(x_{1},\ldots,x_{n})\in I^{n}$, $n\geq2$ and a nonnegative *n*-tuple $\mathbf{p}=(p_{1},\ldots,p_{n})$ is such that $P_{n}=\sum_{i=1}^{n}p_{i}> 0$.

In recent investigations of relation (2) and its numerous consequences, it appeared as a fruitful field for many significant results. We accentuate those which deal with this relation in view of superadditivity and monotonicity of the Jensen-type functionals, in [8, 9] or [10], obtained via [11] and suitably summarized in the monograph [12]. In the following part we are going to make use of relation (2) while presenting certain bounds for a selected spectrum of *f*-divergences that originate from the Csiszár divergence functional.

All of the results thus obtained concerning *f*-divergences are going to be observed here in the context of the Zipf–Mandelbrot law and then specified for the Zipf law.

George Kingsley Zipf (1902–1950) was a linguist after whom one of the most common laws in probability and statistics was named. Today this experimental law for the discrete probability distribution frequently is used in information science, bibliometrics, linguistics, social sciences, economy (where it is known as the Pareto law), as well as in physics, biology, computer science *etc*. Thus the term ‘Zipfian distribution’ is used to describe various types of distributions of the probability occurrences which approximately follow the mathematical form of the Zipf law. It was in the first place established with the frequency of the words in a text in view and as such it revealed a hyperbolic relation. As is *e.g.* explained in [13], if words of a language are sorted in the order of decreasing frequency of usage, a word’s frequency *f* is inversely proportional to its rank *r*, or sequence number in the list and the product of these is a constant: $r\cdot f=C$ (‘A few occur very often while many others occur rarely.’). Benoit Mandelbrot, a mathematician very well known for his contribution in fractal theory, generalized the Zipf law in 1966 [14, 15] according to his field of investigation and gave its improvement for the count of the low-rank words [16]. It is also used in information sciences for the purpose of indexing [17, 18], in ecological field studies [19] and it plays its role in art when determining the aesthetics criteria in music [20]. The Zipf–Mandelbrot law is a discrete probability distribution and is defined by the following probability mass function:

3 $$ f(i;N,s,t)=\frac{1}{(i+t)^{s}H_{N,s, t}}, \quad i=1,\ldots,N, $$

where

4 $$ H_{N,s,t}=\sum_{k=1}^{N} \frac{1}{(k+t)^{s}} $$

is a generalization of a harmonic number and $N\in\{1,2,\ldots\}$, $s> 0$ and $t\in[0, \infty)$ are parameters.

For finite *N* and for $t=0$ the Zipf–Mandelbrot law is simply called the Zipf law. (In particular, if we observe the infinite *N* and $t=0$ we actually have the Zeta distribution.)

According to the expressions above, the probability mass function referring to the Zipf law is

5 $$ f (i; N,s )=\frac{1}{i^{s}\cdot H_{N,s}} \quad\text{where } H_{N,s}=\sum _{k=1}^{N}\frac{1}{k^{s}}. $$

The rest of the paper is organized as follows. In Section 1 we define the Csiszár functional and various *f*-divergences for which we give in Section 3 the results based on relation (2). These are further examined in Section 4 in the light of the Zipf–Mandelbrot law and the Zipf law. For the latter we give in Section 5 a specific application in linguistics, concerning the Kullback–Leibler divergence.

## Preliminaries

The previously mentioned *f*-divergences were studied independently by several matematicians. Here we focus on the Csiszár *f*-divergences. Csiszár [1, 2] introduced the *f*-divergence functional as

6 $$ D_{f}(\mathbf{p},\mathbf{q})=\sum _{i=1}^{n}q_{i}f \biggl(\frac {p_{i}}{q_{i}} \biggr), $$

where $\mathbf{p}=(p_{1},\ldots,p_{n})$ and $\mathbf {q}=(q_{1},\ldots,q_{n})$ are probability distributions, that is, $p_{i}, q_{i}\in[0,1]$, for $i=1,\ldots,n$ with $\sum_{i=1}^{n}p_{i}=\sum_{i=1}^{n}q_{i}=1$ and $f\colon[0,\infty )\rightarrow[0,\infty)$ is a convex function, the so-called ‘distance function’ on the set of all probability distributions.

As in [1], we interpret the undefined expressions by

$$f(0):= \lim_{t\rightarrow0+}f(t);\qquad 0f \biggl(\frac{0}{0} \biggr):=0;\qquad 0f \biggl(\frac{a}{0} \biggr):=\lim_{t\rightarrow0+}tf \biggl( \frac {a}{t} \biggr),\quad a>0. $$

The definition of the *f*-divergence functional (6) can be generalized for a function $f\colon I\rightarrow\mathbb{R}$, $I\subseteq\mathbb{R}$, where $\frac{p_{i}}{q_{i}}\in I$, for every $i=1,\ldots,n$. Since we are going to observe this wider class of functions as well, the corresponding functional (6) will be denoted by $\tilde{D}_{f}(\mathbf{p},\mathbf{q})$ (see also [21]).

The general aspect of the Csiszár divergence functional (6) can be interpreted as a series of well-known entropies, divergencies and distances, for special choices of the kernel *f*. In the sequel we present some of the most frequent among them.

Entropies quantify the diversity, uncertainty and randomness of a system. The concept of the Rényi entropy was introduced by [22] and has been of a great importance in statistics, ecology, theoretical computer science *etc*.

The Rényi entropy of order *α* of **p** is defined as

7 $$ H_{\alpha}(p):=\frac{1}{1-\alpha}\log \Biggl(\sum _{i=1}^{n}p_{i}^{\alpha} \Biggr), $$

where $\alpha\geq0$, $\alpha\neq1$ and $\mathbf{p}=(p_{1},\ldots ,p_{n})$ is a probability distribution. Among special cases of the Rényi entropy (*e.g.* the Hartley or max-entropy, min-entropy, and the collision entropy), the Rényi entropy tends to the Shannon entropy (see [23]) for the limiting value of $\alpha\rightarrow 1$. The Shannon entropy (which is sometimes called information divergence) is thus defined as

8 $$ H(\mathbf{p}):=-\sum_{i=1}^{n}p_{i} \log p_{i}. $$

Besides the absolute entropies, one can also observe the relative entropies, as did Rényi when he introduced a special form of *f*-divergence. The Rényi divergence of order *α*, $\alpha \geq0$, $\alpha\neq1$ for the probability distributions $\mathbf {p}=(p_{1},\ldots,p_{n})$ and $\mathbf{q}=(q_{1},\ldots,q_{n})$ is defined as

9 $$ D_{\alpha}(\mathbf{p},\mathbf{q}):=\frac{1}{\alpha-1}\log \Biggl(\sum_{i=1}^{n}q_{i} \biggl( \frac{p_{i}}{q_{i}} \biggr)^{\alpha } \Biggr). $$

A relation similar to the one between the Rényi entropy and the Shannon entropy holds in the case of the Rényi divergence and the Kullback–Leibler divergence (see [24]) for the probability distributions $\mathbf{p}=(p_{1},\ldots,p_{n})$ and $\mathbf {q}=(q_{1},\ldots,q_{n})$. As $\alpha\rightarrow1$, the Rényi divergence tends to the Kullback–Leibler divergence. The latter is sometimes called the relative entropy and is defined by

10 $$ \mathrm{KL}(\mathbf{p},\mathbf{q}):=\sum_{i=1}^{n}p_{i} \log \biggl(\frac {p_{i}}{q_{i}} \biggr). $$

### Remark 1

Although it is common to take the logarithm function with the base 2, it will not be essential in the sequel. Moreover, we are going to analyze the results including the logarithm function for different (positive) bases, namely, for those greater than 1 as well as for those that are less than 1.

Among various divergences and considering the properties of symmetry and triangular inequality which some of them possess, we can also define certain distances between two probability distributions.

Thus the Hellinger distance between the probability distributions $\mathbf{p}=(p_{1},\ldots,p_{n})$ and $\mathbf{q}=(q_{1},\ldots ,q_{n})$ is defined by

11 $$ h(\mathbf{p},\mathbf{q}):=\frac{1}{\sqrt{2}}\sqrt{\sum _{i=1}^{n} (\sqrt{p_{i}}- \sqrt{q_{i}} )^{2}}. $$

The Hellinger distance is a metric and is often used in its squared form, *i.e.* as $h^{2}(\mathbf{p},\mathbf{q}):=\frac {1}{2}\sum_{i=1}^{n} (\sqrt{p_{i}}-\sqrt{q_{i}} )^{2} $.

Among the values of the order *α* for the Rényi divergence, some have wider application than others. The value 1 is already determined by continuity in *α* since it cannot be calculated directly by (9) and another interesting example is the order $1/2$. This order makes the Rényi divergence symmetric in its arguments. In this context, it is interesting to see how the Rényi divergence, although not itself a metric, relates to the Hellinger distance:

12 $$ D_{1/2}(\mathbf{p},\mathbf{q})=-2\log \biggl(1- \frac{h^{2}(\mathbf {p},\mathbf{q})}{2} \biggr). $$

Furthermore, the Bhattacharyya coefficient is an approximate measure of the amount of overlapping between two distributions and as such can be used to determine their relative closeness. It is defined as

13 $$ B(\mathbf{p},\mathbf{q}):=\sum_{i=1}^{n} \sqrt{p_{i}q_{i}}, $$

whereas the Bhattacharyya distance is defined as $D_{B}(\mathbf {p},\mathbf{q}):=-\log B(\mathbf{p},\mathbf{q})$. The relation between the Bhattacharyya coefficient and the Hellinger distance is

$$h^{2}(\mathbf{p},\mathbf{q})=1- B(\mathbf{p},\mathbf{q}). $$

In order to conclude this overview, let us remind the reader that the $\chi^{2}$ divergence is defined as

14 $$ \chi^{2}(\mathbf{p},\mathbf{q}):=\sum _{i=1}^{n}\frac{ (p_{i}-q_{i} )^{2}}{q_{i}}, $$

the total variation distance or statistical distance is given by

15 $$ V(\mathbf{p},\mathbf{q}):=\sum_{i=1}^{n} \vert p_{i}-q_{i} \vert $$

and the definition of the triangular discrimination reads as follows:

16 $$ \Delta(\mathbf{p},\mathbf{q}):=\sum_{i=1}^{n} \frac{ (p_{i}-q_{i} )^{2}}{p_{i}+q_{i}}. $$

More detailed analyses of the mentioned divergences as well as their wider spectrum one can find *e.g.* in [5, 6, 24].

## Basic relations for *f*-divergences

In order to deduce the relations from relation (2) for the *f*-divergences described in the introductory part, we start with the general result for bounds obtained for the Csiszár functional (6) observed under more general conditions as $\tilde{D}_{f}(\mathbf{p},\mathbf{q})$.

### Theorem 1

*Let* $I\subseteq\mathbb{R}$ *be an interval*. *Suppose* $\mathbf {p}=(p_{1},\ldots,p_{n})$ *is an* *n*-*tuple of real numbers with* $P_{n}=\sum_{i=1}^{n}p_{i}$ *and* $\mathbf{q}=(q_{1},\ldots,q_{n})$ *is an* *n*-*tuple of nonnegative real numbers with* $Q_{n}=\sum_{i=1}^{n}q_{i}$, *such that* $\frac{p_{i}}{q_{i}}\in I$, $i=1,\ldots,n$.

*If* $f \colon I\rightarrow\mathbb{R}$ *is a convex function*, *then*

17 $$\begin{aligned} & \min\{q_{i}\} \Biggl[\sum _{i=1}^{n}f \biggl(\frac {p_{i}}{q_{i}} \biggr)-n f \Biggl(\frac{1}{n}\sum_{i=1}^{n} \frac {p_{i}}{q_{i}} \Biggr) \Biggr]+Q_{n}f \biggl(\frac{P_{n}}{Q_{n}} \biggr) \\ & \quad \leq\tilde{D}_{f}(\mathbf{p},\mathbf {q})\leq\max \{q_{i}\} \Biggl[\sum_{i=1}^{n}f \biggl(\frac {p_{i}}{q_{i}} \biggr)-n f \Biggl(\frac{1}{n}\sum _{i=1}^{n}\frac {p_{i}}{q_{i}} \Biggr) \Biggr]+Q_{n}f \biggl(\frac{P_{n}}{Q_{n}} \biggr). \end{aligned}$$

*If* *f* *is a concave function*, *then the inequality signs are reversed*.

*If* $t\mapsto tf(t)$ *is a convex function*, *then*

18 $$\begin{aligned} & \min\{q_{i}\} \Biggl[\sum _{i=1}^{n}\frac{p_{i}}{q_{i}}f \biggl( \frac{p_{i}}{q_{i}} \biggr)-\sum_{i=1}^{n} \frac{p_{i}}{q_{i}} f \Biggl(\frac{1}{n}\sum_{i=1}^{n} \frac{p_{i}}{q_{i}} \Biggr) \Biggr]+P_{n}f \biggl(\frac{P_{n}}{Q_{n}} \biggr) \\ & \quad \leq\tilde{D}_{\mathrm{id}\cdot f}(\mathbf{p},\mathbf{q})\leq\max \{ q_{i}\} \Biggl[\sum_{i=1}^{n} \frac{p_{i}}{q_{i}}f \biggl(\frac {p_{i}}{q_{i}} \biggr)-\sum _{i=1}^{n} \frac{p_{i}}{q_{i}} f \Biggl( \frac{1}{n}\sum_{i=1}^{n} \frac{p_{i}}{q_{i}} \Biggr) \Biggr]+P_{n}f \biggl(\frac{P_{n}}{Q_{n}} \biggr), \end{aligned}$$

*where* $\tilde{D}_{\mathrm{id}\cdot f}(\mathbf{p},\mathbf{q}):=\sum_{i=1}^{n}p_{i}f (\frac{p_{i}}{q_{i}} )$.

*If* $t\mapsto tf(t)$ *is a concave function*, *then the inequality signs are reversed*.

### Proof

If we observe a convex function *f* and replace $p_{i}$ by $q_{i}$ as well as $x_{i}$ by $\frac{p_{i}}{q_{i}}$ in relation (2), we get (17).

If we then observe the function $t\mapsto tf(t)$ as a convex function and replace $p_{i}$ by $q_{i}$ and $x_{i}$ by $\frac{p_{i}}{q_{i}}$ we get (18). □

The following corollary precedes the related result for the Kullback–Leibler divergence (10). Recall that (10) can be interpreted as a special case of the functional (6).

### Corollary 1

*Let* $\mathbf{p}=(p_{1},\ldots,p_{n})$ *and* $\mathbf{q}=(q_{1},\ldots ,q_{n})$ *be* *n*-*tuples of nonnegative real numbers with* $P_{n}=\sum_{i=1}^{n}p_{i}$ *and* $Q_{n}=\sum_{i=1}^{n}q_{i}$. *Then*

19 $$\begin{aligned} & \min\{q_{i}\} \Biggl[\sum _{i=1}^{n}\frac{p_{i}}{q_{i}} \log\frac {p_{i}}{q_{i}}- \sum_{i=1}^{n}\frac{p_{i}}{q_{i}} \log \Biggl( \frac {1}{n}\sum_{i=1}^{n} \frac{p_{i}}{q_{i}} \Biggr) \Biggr] +P_{n} \log \frac{P_{n}}{Q_{n}} \\ & \quad \leq\sum_{i=1}^{n}p_{i} \log\frac{p_{i}}{q_{i}} \\ & \quad \leq\max\{q_{i}\} \Biggl[\sum _{i=1}^{n}\frac {p_{i}}{q_{i}} \log\frac{p_{i}}{q_{i}}- \sum_{i=1}^{n}\frac {p_{i}}{q_{i}} \log \Biggl( \frac{1}{n}\sum_{i=1}^{n} \frac {p_{i}}{q_{i}} \Biggr) \Biggr] +P_{n} \log\frac {P_{n}}{Q_{n}}, \end{aligned}$$

*where the logarithm base is greater than* 1.

*If the logarithm base is less than* 1, *then the inequality signs are reversed*.

### Proof

It follows from Theorem 1 as a special case of inequalities (18), for the function $t\mapsto t\log t$, which is convex when the logarithm base is greater than 1 (and concave when the base is less than 1). □

If we additionally specify the *n*-tuples **p** and **q** as in the sequel, we provide the bounds for the Kullback–Leibler divergence.

### Remark 2

If we observe $\mathbf{p}=(p_{1},\ldots,p_{n})$ and $\mathbf{q}=(q_{1},\ldots,q_{n})$ as probability distributions, we may write

20 $$\begin{aligned} & \min\{q_{i}\} \Biggl[\sum _{i=1}^{n}\frac{p_{i}}{q_{i}} \log\frac {p_{i}}{q_{i}}- \sum_{i=1}^{n}\frac{p_{i}}{q_{i}} \log \Biggl( \frac {1}{n}\sum_{i=1}^{n} \frac{p_{i}}{q_{i}} \Biggr) \Biggr] \\ & \quad \leq \mathrm{KL}(\mathbf{p},\mathbf{q}) \leq\max\{q_{i}\} \Biggl[\sum_{i=1}^{n}\frac{p_{i}}{q_{i}} \log \frac{p_{i}}{q_{i}}- \sum_{i=1}^{n} \frac{p_{i}}{q_{i}} \log \Biggl(\frac{1}{n}\sum_{i=1}^{n} \frac{p_{i}}{q_{i}} \Biggr) \Biggr], \end{aligned}$$

where the logarithm base is greater than 1.

If the logarithm base is less than 1, then the inequality signs are reversed.

In other words, we obtained the corresponding bounds for the *Kullback–Leibler divergence* (10).

### Remark 3

The Kullback–Leibler divergence is sometimes used in its reversed form $\mathrm{KL}(\mathbf{q},\mathbf{p})$. A similar type of bounds can be obtained when observing the reversed Kullback–Leibler divergence making use of the kernel function $f(t)=-\log t$, its convexity and concavity related to the observed logarithm base (greater than 1 or less than 1, respectively), and following the analogous procedure described in Corollary 1 and Remark 2.

It is natural to observe in a similar fashion the other divergences (distances) described in Section 1: the Hellinger distance, the Bhattacharyya coefficient, the chi-square divergence, the total variation distance and the triangular discrimination.

### Corollary 2

*Let* $\mathbf{p}=(p_{1},\ldots,p_{n})$ *and* $\mathbf{q}=(q_{1},\ldots ,q_{n})$ *be* *n*-*tuples of nonnegative real numbers with* $P_{n}=\sum_{i=1}^{n}p_{i}$ *and* $Q_{n}=\sum_{i=1}^{n}q_{i}$. *Then*

21 $$\begin{aligned} & \frac{1}{2}\min\{q_{i}\} \Biggl[\sum _{i=1}^{n} \biggl(1-\sqrt { \frac{p_{i}}{q_{i}}} \biggr)^{2}-n \Biggl(1-\sqrt{ \frac{1}{n}\sum_{i=1}^{n} \frac{p_{i}}{q_{i}}} \Biggr)^{2} \Biggr] +Q_{n} \biggl(1-\sqrt{ \frac{P_{n}}{Q_{n}}} \biggr)^{2} \\ & \quad \leq\frac{1}{2}\sum_{i=1}^{n} (\sqrt {p_{i}}-\sqrt{q_{i}} )^{2} \\ & \quad \leq\frac{1}{2}\max\{q_{i}\} \Biggl[\sum _{i=1}^{n} \biggl(1-\sqrt{\frac{p_{i}}{q_{i}}} \biggr)^{2}-n \Biggl(1-\sqrt{\frac{1}{n}\sum _{i=1}^{n}\frac{p_{i}}{q_{i}}} \Biggr)^{2} \Biggr]+Q_{n} \biggl(1-\sqrt{\frac{P_{n}}{Q_{n}}} \biggr)^{2}. \end{aligned}$$

### Proof

It follows from Theorem 1 as a special case of inequalities (17), for the convex function $t\rightarrow\frac{1}{2} (\sqrt{t}-1 )^{2}$. □

### Remark 4

If we observe $\mathbf{p}=(p_{1},\ldots,p_{n})$ and $\mathbf{q}=(q_{1},\ldots,q_{n})$ as probability distributions, we may write

22 $$\begin{aligned} & \frac{1}{2}\min\{q_{i}\} \Biggl[\sum _{i=1}^{n} \biggl(1-\sqrt { \frac{p_{i}}{q_{i}}} \biggr)^{2}-n \Biggl(1-\sqrt{ \frac{1}{n}\sum_{i=1}^{n} \frac{p_{i}}{q_{i}}} \Biggr)^{2} \Biggr] \\ & \quad \leq h^{2}(\mathbf{p},\mathbf{q}) \\ & \quad \leq\frac{1}{2}\max\{q_{i}\} \Biggl[\sum _{i=1}^{n} \biggl(1-\sqrt{\frac{p_{i}}{q_{i}}} \biggr)^{2}-n \Biggl(1-\sqrt{\frac{1}{n}\sum _{i=1}^{n}\frac{p_{i}}{q_{i}}} \Biggr)^{2} \Biggr]. \end{aligned}$$

In other words, we obtained the corresponding bounds for the *(squared) Hellinger distance* $h^{2}(\mathbf {p},\mathbf{q})$.

### Corollary 3

*Let* $\mathbf{p}=(p_{1},\ldots,p_{n})$ *and* $\mathbf{q}=(q_{1},\ldots ,q_{n})$ *be* *n*-*tuples of nonnegative real numbers with* $P_{n}=\sum_{i=1}^{n}p_{i}$ *and* $Q_{n}=\sum_{i=1}^{n}q_{i}$. *Then*

23 $$\begin{aligned} & \min\{q_{i}\} \Biggl[\sqrt{n\sum _{i=1}^{n}\frac {p_{i}}{q_{i}}}-\sum _{i=1}^{n}\sqrt{\frac{p_{i}}{q_{i}}} \Biggr]- \sqrt{P_{n}Q_{n}} \\ & \quad \leq- \sum_{i=1}^{n} \sqrt{p_{i}q_{i}} \\ & \quad \leq\max\{q_{i}\} \Biggl[\sqrt{n\sum _{i=1}^{n}\frac{p_{i}}{q_{i}}}-\sum _{i=1}^{n}\sqrt{\frac {p_{i}}{q_{i}}} \Biggr]- \sqrt{P_{n}Q_{n}}. \end{aligned}$$

### Proof

It follows from Theorem 1 as a special case of inequalities (17), for the convex function $f(t)=-\sqrt{t}$. □

### Remark 5

If we observe $\mathbf{p}=(p_{1},\ldots,p_{n})$ and $\mathbf{q}=(q_{1},\ldots,q_{n})$ as probability distributions and adopt by the definition (13) that $B(\mathbf {p},\mathbf{q})=\sqrt{p_{i}q_{i}}$, we may write

24 $$\begin{aligned} & \min\{q_{i}\} \Biggl[\sqrt{n\sum _{i=1}^{n}\frac {p_{i}}{q_{i}}}-\sum _{i=1}^{n}\sqrt{\frac{p_{i}}{q_{i}}} \Biggr]-1 \\ & \quad \leq-B(\mathbf{p},\mathbf{q}) \\ & \quad\leq \max\{q_{i}\} \leq \Biggl[\sqrt{n\sum _{i=1}^{n}\frac{p_{i}}{q_{i}}}-\sum _{i=1}^{n}\sqrt{\frac {p_{i}}{q_{i}}} \Biggr]-1, \end{aligned}$$

or

25 $$\begin{aligned} & 1-\min\{q_{i}\} \Biggl[\sqrt{n\sum _{i=1}^{n}\frac {p_{i}}{q_{i}}}-\sum _{i=1}^{n}\sqrt{\frac{p_{i}}{q_{i}}} \Biggr] \\ & \quad \geq B(\mathbf{p},\mathbf{q}) \geq1-\max\{q_{i}\} \Biggl[\sqrt{n\sum _{i=1}^{n}\frac{p_{i}}{q_{i}}}-\sum _{i=1}^{n}\sqrt{\frac {p_{i}}{q_{i}}} \Biggr]. \end{aligned}$$

In other words, we obtained the corresponding bounds for the *Bhattacharyya coefficient* $B(\mathbf{p},\mathbf {q})$.

### Corollary 4

*Let* $\mathbf{p}=(p_{1},\ldots,p_{n})$ *be an* *n*-*tuple of real numbers and* $\mathbf{q}=(q_{1},\ldots,q_{n})$ *an* *n*-*tuple of nonnegative real numbers with* $P_{n}=\sum_{i=1}^{n}p_{i}$ *and* $Q_{n}=\sum_{i=1}^{n}q_{i}$. *Then*

26 $$\begin{aligned} & \min\{q_{i}\} \Biggl[\sum _{i=1}^{n} \biggl(\frac {p_{i}}{q_{i}}-1 \biggr)^{2}-n \Biggl(\frac{1}{n}\sum_{i=1}^{n} \frac {p_{i}}{q_{i}}-1 \Biggr)^{2} \Biggr] +\frac{ (P_{n}-Q_{n} )^{2}}{Q_{n}} \\ & \quad \leq\sum_{i=1}^{n} \frac{ (p_{i}-q_{i} )^{2}}{q_{i}} \\ & \quad \leq\max\{q_{i}\} \Biggl[\sum _{i=1}^{n} \biggl(\frac{p_{i}}{q_{i}}-1 \biggr)^{2}-n \Biggl(\frac{1}{n}\sum_{i=1}^{n} \frac{p_{i}}{q_{i}}-1 \Biggr)^{2} \Biggr] +\frac{ (P_{n}-Q_{n} )^{2}}{Q_{n}}. \end{aligned}$$

### Proof

It follows from Theorem 1 as a special case of inequalities (17), for the convex function $f(t)=(t-1)^{2}$. □

### Remark 6

If we observe $\mathbf{p}=(p_{1},\ldots,p_{n})$ and $\mathbf{q}=(q_{1},\ldots,q_{n})$ as probability distributions, we may write

27 $$\begin{aligned} & \min\{q_{i}\} \Biggl[\sum _{i=1}^{n} \biggl(\frac {p_{i}}{q_{i}}-1 \biggr)^{2}-n \Biggl(\frac{1}{n}\sum_{i=1}^{n} \frac {p_{i}}{q_{i}}-1 \Biggr)^{2} \Biggr] \\ & \quad \leq\chi^{2}(\mathbf{p},\mathbf{q}) \\ & \quad \leq\max\{q_{i}\} \Biggl[\sum _{i=1}^{n} \biggl(\frac{p_{i}}{q_{i}}-1 \biggr)^{2}-n \Biggl(\frac{1}{n}\sum_{i=1}^{n} \frac{p_{i}}{q_{i}}-1 \Biggr)^{2} \Biggr]. \end{aligned}$$

In other words, we obtained the corresponding bounds for the *chi-square divergence* $\chi^{2}(\mathbf {p},\mathbf{q})$.

### Corollary 5

*Let* $\mathbf{p}=(p_{1},\ldots,p_{n})$ *be an* *n*-*tuple of real numbers and* $\mathbf{q}=(q_{1},\ldots,q_{n})$ *an* *n*-*tuple of nonnegative real numbers with* $P_{n}=\sum_{i=1}^{n}p_{i}$ *and* $Q_{n}=\sum_{i=1}^{n}q_{i}$. *Then*

28 $$\begin{aligned} & \min\{q_{i}\} \Biggl[\sum _{i=1}^{n} \biggl\vert \frac {p_{i}}{q_{i}}-1 \biggr\vert -n \Biggl\vert \frac{1}{n}\sum_{i=1}^{n} \frac {p_{i}}{q_{i}}-1 \Biggr\vert \Biggr] + \vert P_{n}-Q_{n} \vert \\ &\quad \leq\sum_{i=1}^{n} \vert p_{i}-q_{i} \vert \\ & \quad \leq\max\{q_{i}\} \Biggl[\sum _{i=1}^{n} \biggl\vert \frac{p_{i}}{q_{i}}-1 \biggr\vert -n \Biggl\vert \frac{1}{n}\sum_{i=1}^{n} \frac{p_{i}}{q_{i}}-1 \Biggr\vert \Biggr] + \vert P_{n}-Q_{n} \vert . \end{aligned}$$

### Proof

It follows from Theorem 1 as a special case of inequalities (17), for the convex function $f(t)= \vert t-1 \vert $. □

### Remark 7

If we observe $\mathbf{p}=(p_{1},\ldots,p_{n})$ and $\mathbf{q}=(q_{1},\ldots,q_{n})$ as probability distributions, we may write

29 $$\begin{aligned} & \min\{q_{i}\} \Biggl[\sum _{i=1}^{n} \biggl\vert \frac {p_{i}}{q_{i}}-1 \biggr\vert -n \Biggl\vert \frac{1}{n}\sum_{i=1}^{n} \frac {p_{i}}{q_{i}}-1 \Biggr\vert \Biggr] \\ & \quad \leq V(\mathbf{p},\mathbf{q}) \\ & \quad \leq\max\{q_{i}\} \Biggl[\sum _{i=1}^{n} \biggl\vert \frac{p_{i}}{q_{i}}-1 \biggr\vert -n \Biggl\vert \frac{1}{n}\sum_{i=1}^{n} \frac{p_{i}}{q_{i}}-1 \Biggr\vert \Biggr]. \end{aligned}$$

In other words, we obtained the corresponding bounds for the *total variation distance* $V(\mathbf{p},\mathbf {q})$.

### Corollary 6

*Let* $\mathbf{p}=(p_{1},\ldots,p_{n})$ *be an* *n*-*tuple of real numbers and* $\mathbf{q}=(q_{1},\ldots,q_{n})$ *an* *n*-*tuple of nonnegative real numbers with* $P_{n}=\sum_{i=1}^{n}p_{i}$ *and* $Q_{n}=\sum_{i=1}^{n}q_{i}$. *Then*

30 $$\begin{aligned} & \min\{q_{i}\} \Biggl[\sum _{i=1}^{n}\frac {(p_{i}-q_{i})^{2}}{q_{i}(p_{i}+q_{i})}-n \frac{ (\frac{1}{n} \sum_{i=1}^{n}\frac{p_{i}}{q_{i}}-1 )^{2}}{\frac{1}{n}\sum_{i=1}^{n}\frac{p_{i}}{q_{i}}+1} \Biggr]+\frac {(P_{n}-Q_{n})^{2}}{P_{n}+Q_{n}} \\ & \quad \leq\sum_{i=1}^{n} \frac {(p_{i}-q_{i})^{2}}{p_{i}+q_{i}} \\ & \quad \leq\max\{q_{i}\} \Biggl[\sum _{i=1}^{n}\frac {(p_{i}-q_{i})^{2}}{q_{i}(p_{i}+q_{i})}-n \frac{ (\frac{1}{n} \sum_{i=1}^{n}\frac{p_{i}}{q_{i}}-1 )^{2}}{\frac{1}{n}\sum_{i=1}^{n}\frac{p_{i}}{q_{i}}+1} \Biggr]+\frac {(P_{n}-Q_{n})^{2}}{P_{n}+Q_{n}}. \end{aligned}$$

### Proof

It follows from Theorem 1 as a special case of inequalities (17), for the convex function $f(t)=\frac {(t-1)^{2}}{t+1}$. □

### Remark 8

If we observe $\mathbf{p}=(p_{1},\ldots,p_{n})$ and $\mathbf{q}=(q_{1},\ldots,q_{n})$ as probability distributions, we may write

31 $$\begin{aligned} & \min\{q_{i}\} \Biggl[\sum _{i=1}^{n}\frac {(p_{i}-q_{i})^{2}}{q_{i}(p_{i}+q_{i})}-n \frac{ (\frac{1}{n} \sum_{i=1}^{n}\frac{p_{i}}{q_{i}}-1 )^{2}}{\frac{1}{n}\sum_{i=1}^{n}\frac{p_{i}}{q_{i}}+1} \Biggr] \\ & \quad \leq\Delta(\mathbf{p},\mathbf{q}) \\ & \quad \leq\max\{q_{i}\} \Biggl[\sum _{i=1}^{n}\frac {(p_{i}-q_{i})^{2}}{q_{i}(p_{i}+q_{i})}-n \frac{ (\frac{1}{n} \sum_{i=1}^{n}\frac{p_{i}}{q_{i}}-1 )^{2}}{\frac{1}{n}\sum_{i=1}^{n}\frac{p_{i}}{q_{i}}+1} \Biggr]. \end{aligned}$$

In other words, we obtained the corresponding bounds for the *triangular discrimination* $\Delta(\mathbf {p},\mathbf{q})$.

## On *f*-divergences for the Zipf–Mandelbrot law

In this section we are going to derive the results from the previous section for the Zipf–Mandelbrot law (3). Namely, if we put $q_{i}=f(i;N,s,t)$ in (3) as its probability mass function, we can observe obtained results in the light of the Zipf–Mandelbrot law.

For this purpose we present the general results concerning the Csiszár functional $\tilde{D}_{f}(\mathbf{p},\mathbf{q})$ for the Zipf–Mandelbrot law. If we define **q** via (3) as a Zipf–Mandelbrot law *N*-tuple, the definition (6) of the Csiszár functional becomes

32 $$ \tilde{D}_{f}(i, N,s_{2},t_{2}, \mathbf{p})= \sum_{i=1}^{N} \frac {1}{ (i+t_{2} )^{s_{2}}H_{N,s_{2},t_{2}}}f \bigl(p_{i} (i+t_{2} )^{s_{2}}H_{N,s_{2},t_{2}} \bigr), $$

where $f \colon I\rightarrow\mathbb{R}$, $I\subseteq\mathbb{R}$, and the parameters $N \in\mathbb{N}$, $s_{2}> 0$, $t_{2}\geq0$ are such that $p_{i} (i+t_{2} )^{s_{2}}H_{N,s_{2},t_{2}}\in I$, $i=1,\ldots,N$.

The Csiszár functional (6) assumes the following form when **p** and **q** are both defined as Zipf–Mandelbrot law *N*-tuples:

33 $$ \tilde{D}_{f}(i, N,s_{1},s_{2},t_{1}, t_{2})= \sum_{i=1}^{N} \frac {1}{(i+t_{2})^{s_{2}}H_{N,s_{2},t_{2}}}f \biggl(\frac{ (i+t_{2} )^{s_{2}}H_{N,s_{2},t_{2}}}{ (i+t_{1} )^{s_{1}}H_{N,s_{1},t_{1}}} \biggr), $$

where $f \colon I\rightarrow\mathbb{R}$, $I\subseteq\mathbb{R}$, and $N \in\mathbb{N}$, $s_{1}, s_{2}> 0$, $t_{1},t_{2}\geq0$ are such that $\frac{ (i+t_{2} )^{s_{2}}H_{N,s_{2},t_{2}}}{ (i+t_{1} )^{s_{1}}H_{N,s_{1},t_{1}}}\in I$, $i=1,\ldots,N$.

Finally, both **p** and **q** *N*-tuples may be defined via the Zipf law (5) and thus the Csiszár functional (6) assumes the form

34 $$ \tilde{D}_{f}(i, N,s_{1},s_{2})= \sum_{i=1}^{N}\frac {1}{i^{s_{2}}H_{N,s_{2}}}f \biggl(i^{s_{2}-s_{1}}\frac {H_{N,s_{2}}}{H_{N,s_{1}}} \biggr). $$

Our next step is providing the corresponding forms of Theorem 1 which are suitable for further applications. Thus we start with the Csiszár functional $\tilde{D}_{f}(i, N,s_{2},t_{2},\mathbf{p})$, which implies single Zipf–Mandelbrot laws $q_{i}$, for $i=1,\ldots,N$.

### Corollary 7

*Let* $\mathbf{p}=(p_{1},\ldots,p_{N})$ *be an* *N*-*tuple of real numbers with* $P_{N}=\sum_{i=1}^{N}p_{i}$. *Suppose* $I\subseteq\mathbb{R}$ *is an interval*, $N \in\mathbb{N}$ *and* $s_{2}> 0$, $t_{2}\geq0$ *are such that* $p_{i}(i+t_{2})^{s_{2}}H_{N,s_{2},t_{2}}\in I$, $i=1,\ldots ,N$.

*If* $f \colon I\rightarrow\mathbb{R}$ *is a convex function*, *then*

35 $$\begin{aligned} \frac{1}{(N+t_{2})^{s_{2}}H_{N,s_{2},t_{2}}}\Lambda_{1}+P_{N}f(P_{N}) &\leq\tilde{D}_{f}(i, N,s_{2}, t_{2},\mathbf{p}) \\ &\leq \frac{1}{(1+t_{2})^{s_{2}}H_{N,s_{2},t_{2}}}\Lambda _{1}+P_{N}f(P_{N}), \end{aligned}$$

*where*

$$\begin{aligned} \Lambda_{1}=\sum_{i=1}^{N}f \bigl(p_{i}(i+t_{2})^{s_{2}}H_{N,s_{2},t_{2}} \bigr)- Nf \Biggl(\frac{1}{N}\sum_{i=1}^{N}p_{i}(i+t_{2})^{s_{2}}H_{N,s_{2},t_{2}} \Biggr). \end{aligned}$$

*If* *f* *is a concave function*, *then the inequality signs are reversed*.

*If* $t\mapsto tf(t)$ *is a convex function*, *then*

36 $$\begin{aligned} \frac{1}{(N+t_{2})^{s_{2}}H_{N,s_{2},t_{2}}}\bar{\Lambda }_{1}+P_{N}f(P_{N}) &\leq\tilde{D}_{\mathrm{id}\cdot f}(i, N, s_{2},t_{2},\mathbf{p}) \\ &\leq\frac {1}{(1+t_{2})^{s_{2}}H_{N,s_{2},t_{2}}}\bar{\Lambda}_{1}+P_{N}f(P_{N}), \end{aligned}$$

*where* $\tilde{D}_{\mathrm{id}\cdot f}(i, N, s_{2},t_{2},\mathbf{p}):=\sum_{i=1}^{N}p_{i}f (p_{i}(i+t_{2})^{s_{2}}H_{N,s_{2},t_{2}} )$ *and*

$$\begin{aligned} \bar{\Lambda}_{1}={}&\sum_{i=1}^{N}p_{i}(i+t_{2})^{s_{2}}H_{N,s_{2},t_{2}}f \bigl(p_{i}(i+t_{2})^{s_{2}}H_{N,s_{2},t_{2}} \bigr) \\ & {} -\sum_{i=1}^{N}p_{i}(i+t_{2})^{s_{2}}H_{N,s_{2},t_{2}}f \Biggl(\frac {1}{N}\sum_{i=1}^{N}p_{i}(i+t_{2})^{s_{2}}H_{N,s_{2},t_{2}} \Biggr). \end{aligned}$$

*If* $t\mapsto tf(t)$ *is a concave function*, *then the inequality signs are reversed*.

### Proof

It leans on the proof of Theorem 1 with its described substitutions, where we insert for $q_{i}$ the expression $\frac{1}{(i+t_{2})^{s_{2}}H_{N,s_{2},t_{2}}}, i=1,\ldots,N$, which defines the Zipf–Mandelbrot law (3), with $Q_{N}=1$. Since the minimal value for $q_{i}$ is $\min\{q_{i}\}= \frac {1}{(N+t_{2})^{s_{2}}H_{N,s_{2},t_{2}}}$ and its maximal value is $\max \{q_{i}\}= \frac{1}{(1+t_{2})^{s_{2}}H_{N,s_{2},t_{2}}}$, inequalities (35) and (36) follow for the convex functions *f* and $t\mapsto tf(t)$, respectively. They change their signs in the case of concavity as a consequence of the Jensen inequality implicitly included. □

If we have both **p** and **q** defined via the Zipf–Mandelbrot law, then the following corollary plays a role.

### Corollary 8

*Let* $I\subseteq\mathbb{R}$ *be an interval and suppose* $N \in\mathbb {N}$, $s_{1}, s_{2}> 0$, $t_{1},t_{2}\geq0$ *are such that* $\frac { (i+t_{2} )^{s_{2}}H_{N,s_{2},t_{2}}}{ (i+t_{1} )^{s_{1}}H_{N,s_{1},t_{1}}}\in I$, $i=1,\ldots,N$.

*If* $f \colon I\rightarrow\mathbb{R}$ *is a convex function*, *then*

37 $$\begin{aligned} \frac{1}{(N+t_{2})^{s_{2}}H_{N,s_{2},t_{2}}}\Lambda_{2}+f(1)\leq \tilde{D}_{f}(i, N,s_{1},s_{2}, t_{1},t_{2}) \leq\frac {1}{(1+t_{2})^{s_{2}}H_{N,s_{2},t_{2}}}\Lambda_{2}+f(1), \end{aligned}$$

*where*

$$\begin{aligned} \Lambda_{2}=\sum_{i=1}^{N}f \biggl(\frac {(i+t_{2})^{s_{2}}H_{N,s_{2},t_{2}}}{(i+t_{1})^{s_{1}}H_{N,s_{1},t_{1}}} \biggr)- Nf \Biggl(\frac{1}{N}\sum _{i=1}^{N}\frac {(i+t_{2})^{s_{2}}H_{N,s_{2},t_{2}}}{(i+t_{1})^{s_{1}}H_{N,s_{1},t_{1}}} \Biggr). \end{aligned}$$

*If* *f* *is a concave function*, *then the inequality signs are reversed*.

*If* $t\mapsto tf(t)$ *is a convex function*, *then*

38 $$\begin{aligned} \frac{1}{(N+t_{2})^{s_{2}}H_{N,s_{2},t_{2}}}\bar{\Lambda}_{2}+f(1) \leq \tilde{D}_{\mathrm{id}\cdot f}(i, N, s_{1},s_{2},t_{1},t_{2}) \leq\frac {1}{(1+t_{2})^{s_{2}}H_{N,s_{2},t_{2}}}\bar{\Lambda}_{2}+f(1), \end{aligned}$$

*where* $\tilde{D}_{\mathrm{id}\cdot f}(i, N, s_{1},s_{2},t_{1},t_{2}):=\sum_{i=1}^{N}\frac{1}{(i+t_{1})^{s_{1}}H_{N,s_{1},t_{1}}}f (\frac {(i+t_{2})^{s_{2}}H_{N,s_{2},t_{2}}}{(i+t_{1})^{s_{1}}H_{N,s_{1},t_{1}}} )$ *and*

$$\begin{aligned} \bar{\Lambda}_{2}={}&\sum_{i=1}^{N} \frac {(i+t_{2})^{s_{2}}H_{N,s_{2},t_{2}}}{(i+t_{1})^{s_{1}}H_{N,s_{1},t_{1}}} f \biggl(\frac {(i+t_{2})^{s_{2}}H_{N,s_{2},t_{2}}}{(i+t_{1})^{s_{1}}H_{N,s_{1},t_{1}}} \biggr) \\ & {} -\sum_{i=1}^{N} \frac {(i+t_{2})^{s_{2}}H_{N,s_{2},t_{2}}}{(i+t_{1})^{s_{1}}H_{N,s_{1},t_{1}}} f \Biggl(\frac{1}{N}\sum_{i=1}^{N} \frac {(i+t_{2})^{s_{2}}H_{N,s_{2},t_{2}}}{(i+t_{1})^{s_{1}}H_{N,s_{1},t_{1}}} \Biggr). \end{aligned}$$

*If* $t\mapsto tf(t)$ *is a concave function*, *then the inequality signs are reversed*.

### Proof

Since the corollary is a special case of the previous one, its proof is provided by inserting equation (3), which defines the Zipf–Mandelbrot law instead of $p_{i}$, $i=1,\ldots,N$, as was already done for $q_{i}$. That is, $p_{i}= \frac {1}{(i+t_{1})^{s_{1}}H_{N,s_{1},t_{1}}}, i=1,\ldots,N$, where $P_{N}=1$. The rest of the proof follows along the same lines as in Corollary 7, so inequalities (37) and (38) follow for convex functions *f* and $t\mapsto tf(t)$, respectively. They change their signs in the case of concavity as a consequence of the Jensen inequality implicitly included. □

Finally, if both **p** and **q** are defined via the Zipf law (5), then the following statements hold.

### Corollary 9

*Let* $I\subseteq\mathbb{R}$ *be an interval and suppose* $N \in\mathbb {N}$, $s_{1}, s_{2}> 0$ *are such that* $i^{s_{2}-s_{1}}\frac {H_{N,s_{2}}}{H_{N,s_{1}}}\in I$, $i=1,\ldots,N$.

*If* $f \colon I\rightarrow\mathbb{R}$ *is a convex function*, *then*

39 $$\begin{aligned} \frac{1}{N^{s_{2}}H_{N,s_{2}}}\Lambda_{3}+f(1) \leq \tilde{D}_{f}(i, N,s_{1},s_{2})\leq \frac{1}{H_{N,s_{2}}}\Lambda_{3}+f(1), \end{aligned}$$

*where*

$$\begin{aligned} \Lambda_{3}=\sum_{i=1}^{N}f \biggl(i^{s_{2}-s_{1}}\frac {H_{N,s_{2}}}{H_{N,s_{1}}} \biggr)- Nf \Biggl(\frac{1}{N} \sum_{i=1}^{N}i^{s_{2}-s_{1}} \frac {H_{N,s_{2}}}{H_{N,s_{1}}} \Biggr). \end{aligned}$$

*If* *f* *is a concave function*, *then the inequality signs are reversed*.

*If* $t\mapsto tf(t)$ *is a convex function*, *then*

40 $$\begin{aligned} \frac{1}{N^{s_{2}}H_{N,s_{2}}}\bar{\Lambda}_{3}+f(1) \leq \tilde{D}_{\mathrm{id}\cdot f}(i, N, s_{1},s_{2})\leq \frac {1}{H_{N,s_{2}}}\bar{\Lambda}_{3}+f(1), \end{aligned}$$

*where* $\tilde{D}_{\mathrm{id}\cdot f}(i, N, s_{1},s_{2}):=\sum_{i=1}^{N}\frac {1}{i^{s_{2}}H_{N,s_{2}}}f (i^{s_{2}-s_{1}}\frac {H_{N,s_{2}}}{H_{N,s_{1}}} )$ *and*

$$\begin{aligned} \bar{\Lambda}_{3}=\sum_{i=1}^{N}i^{s_{2}-s_{1}} \frac {H_{N,s_{2}}}{H_{N,s_{1}}} f \biggl(i^{s_{2}-s_{1}}\frac{H_{N,s_{2}}}{H_{N,s_{1}}} \biggr) -\sum _{i=1}^{N}i^{s_{2}-s_{1}}\frac{H_{N,s_{2}}}{H_{N,s_{1}}} f \Biggl(\frac{1}{N}\sum_{i=1}^{N}i^{s_{2}-s_{1}} \frac {H_{N,s_{2}}}{H_{N,s_{1}}} \Biggr). \end{aligned}$$

*If* $t\mapsto tf(t)$ *is a concave function*, *then the inequality signs are reversed*.

### Proof

Inequalities (39) and (40) are proved analogously to Corollary 8 if we observe the probability mass functions $p_{i}$ and $q_{i}$ as Zipf laws defined by (5). □

Let us provide the accompanied results of this type for some special cases of *f*-divergences, starting with the Kullback–Leibler divergence (10). Again, we firstly observe the more general case in which only one of two *N*-tuples **p** and **q** is defined via the Zipf–Mandelbrot law (3).

### Corollary 10

*Let* $\mathbf{p}=(p_{1},\ldots,p_{N})$ *be an* *N*-*tuple of nonnegative real numbers with* $P_{N}=\sum_{i=1}^{N}p_{i}$, $N \in\mathbb{N}$ *and* $s_{2}> 0$, $t_{2}\geq0$.

*If the logarithm base is greater than* 1, *then*

41 $$ \frac{1}{(N+t_{2})^{s_{2}}H_{N,s_{2},t_{2}}} \Lambda^{1}_{\mathrm{KL}}\leq \mathrm{KL}(i, N, s_{2},t_{2},\mathbf{p})\leq\frac {1}{(1+t_{2})^{s_{2}}H_{N,s_{2},t_{2}}} \Lambda^{1}_{\mathrm{KL}}, $$

*where*

$$\begin{aligned} \Lambda^{1}_{\mathrm{KL}}={}&\sum_{i=1}^{N}p_{i}(i+t_{2})^{s_{2}} H_{N,s_{2},t_{2}} \log \bigl(p_{i}(i+t_{2})^{s_{2}} H_{N,s_{2},t_{2}} \bigr) \\ & {} -\sum_{i=1}^{N}p_{i}(i+t_{2})^{s_{2}} H_{N,s_{2},t_{2}} \log \Biggl(\frac {1}{N}\sum_{1=1}^{N}p_{i}(i+t_{2})^{s_{2}} H_{N,s_{2},t_{2}} \Biggr). \end{aligned}$$

*If the logarithm base is less than* 1, *then the inequality signs are reversed*.

### Proof

It follows from Corollary 7 as a special case of inequalities (36), for the function $t\mapsto t\log t$, which is convex when the logarithm base is greater than 1. It can also be derived from Corollary 1 and Remark 2 in the context of the Zipf–Mandelbrot law. □

When both **p** and **q** are defined via the Zipf–Mandelbrot law (3) or via the Zipf law (5), the following statements hold.

### Corollary 11

*Let* $N \in\mathbb{N}$ *and* $s_{1}, s_{2}> 0$, $t_{1},t_{2}\geq0$.

*If the logarithm base is greater than* 1, *then*

42 $$ \frac{1}{(N+t_{2})^{s_{2}}H_{N,s_{2},t_{2}}} \Lambda^{2}_{\mathrm{KL}}\leq \mathrm{KL}(i, N, s_{1}, s_{2}, t_{1},t_{2})\leq \frac {1}{(1+t_{2})^{s_{2}}H_{N,s_{2},t_{2}}} \Lambda^{2}_{\mathrm{KL}}, $$

*where*

$$\begin{aligned} \Lambda^{2}_{\mathrm{KL}}={}&\sum_{i=1}^{N} \frac{(i+t_{2})^{s_{2}} H_{N,s_{2},t_{2}}}{(i+t_{1})^{s_{1}} H_{N,s_{1},t_{1}}} \log \biggl(\frac{(i+t_{2})^{s_{2}} H_{N,s_{2},t_{2}}}{(i+t_{1})^{s_{1}} H_{N,s_{1},t_{1}}} \biggr) \\ & {} -\sum_{i=1}^{N} \frac{(i+t_{2})^{s_{2}} H_{N,s_{2},t_{2}}}{(i+t_{1})^{s_{1}} H_{N,s_{1},t_{1}}} \log \Biggl(\frac{1}{N}\sum _{1=1}^{N}\frac {(i+t_{2})^{s_{2}} H_{N,s_{2},t_{2}}}{(i+t_{1})^{s_{1}} H_{N,s_{1},t_{1}}} \Biggr). \end{aligned}$$

*If the parameters* $t_{1},t_{2}=0$, *the corresponding inequalities for the Zipf law follow*:

43 $$ \frac{1}{N^{s_{2}}H_{N,s_{2}}} \tilde{\Lambda}^{2}_{\mathrm{KL}} \leq \mathrm{KL}(i, N, s_{1}, s_{2})\leq\frac{1}{H_{N,s_{2}}} \tilde{ \Lambda}^{2}_{\mathrm{KL}}, $$

*where*

$$\begin{aligned} \tilde{\Lambda}^{2}_{\mathrm{KL}}={}&\sum _{i=1}^{N}\frac{i^{s_{2}-s_{1}} H_{N,s_{2}}}{H_{N,s_{1}}} \log \biggl( \frac{i^{s_{2}-s_{1}} H_{N,s_{2}}}{H_{N,s_{1}}} \biggr) \\ & {} -\sum_{i=1}^{N} \frac{i^{s_{2}-s_{1}} H_{N,s_{2}}}{H_{N,s_{1}}} \log \Biggl(\frac{1}{N}\sum _{1=1}^{N}\frac{i^{s_{2}-s_{1}} H_{N,s_{2}}}{H_{N,s_{1}}} \Biggr). \end{aligned}$$

*If the logarithm base is less than* 1, *then the signs in inequalities* (42) *and* (43) *are reversed*.

### Proof

Inequalities (42) follow from Corollary 8 as a special case of inequalities (38), for the function $t\mapsto t\log t$, which is convex when the logarithm base is greater than 1.

Similarly, inequalities (43) follow from Corollary 9 as a special case of inequalities (40). □

The following corollaries deal with the Hellinger distance (11) considering one or two *N*-tuples defined via the Zipf–Mandelbrot law or the Zipf law, as its special case.

### Corollary 12

*Let* $\mathbf{p}=(p_{1},\ldots,p_{N})$ *be an* *N*-*tuple of nonnegative real numbers with* $P_{N}=\sum_{i=1}^{N}p_{i}$, $N \in\mathbb{N}$ *and* $s_{2}> 0$, $t_{2}\geq0$.

*Then*

44 $$ \frac{1}{2(N+t_{2})^{s_{2}}H_{N,s_{2},t_{2}}} \Lambda^{1}_{h}\leq h^{2}(i, N, s_{2},t_{2},\mathbf{p})\leq \frac {1}{2(1+t_{2})^{s_{2}}H_{N,s_{2},t_{2}}} \Lambda^{1}_{h}, $$

*where*

$$\Lambda_{h}^{1}=\sum_{i=1}^{N}{\left(1-\sqrt{p_{i}{\left(i+t_{2}\right)}^{s_{2}}H_{N,s_{2},t_{2}}}\right)}^{2}-N{\left(1-\sqrt{\frac{1}{N}\sum_{i=1}^{N}p_{i}{\left(i+t_{2}\right)}^{s_{2}}H_{N,s_{2},t_{2}}}\right)}^{2}.$$

### Proof

It follows from Corollary 7 as a special case of inequalities (35), for the convex function $t\mapsto\frac{1}{2} (\sqrt{t}-1 )^{2}$. It can also be derived from Corollary 2 and Remark 4 in the context of the Zipf–Mandelbrot law. □

When both **p** and **q** are defined via the Zipf–Mandelbrot law (3) or via the Zipf law (5), the following statements hold.

### Corollary 13

*Let* $N \in\mathbb{N}$ *and* $s_{1}, s_{2}> 0$, $t_{1},t_{2}\geq0$.

*Then*

45 $$ \frac{1}{2(N+t_{2})^{s_{2}}H_{N,s_{2},t_{2}}} \Lambda^{2}_{h}\leq h^{2}(i, N, s_{1}, s_{2}, t_{1},t_{2}) \leq\frac {1}{2(1+t_{2})^{s_{2}}H_{N,s_{2},t_{2}}} \Lambda^{2}_{h}, $$

*where*

$$\begin{aligned} \Lambda^{2}_{h}=\sum_{i=1}^{N} \biggl(1-\sqrt{\frac{ (i+t_{1} )^{s_{1}}H_{N,s_{1},t_{1}}}{ (i+t_{2} )^{s_{2}}H_{N,s_{2},t_{2}}} } \biggr)^{2}-N \Biggl(1-\sqrt {\frac {1}{N}\sum_{i=1}^{N} \frac{ (i+t_{1} )^{s_{1}}H_{N,s_{1},t_{1}}}{ (i+t_{2} )^{s_{2}}H_{N,s_{2},t_{2}}}} \Biggr)^{2}. \end{aligned}$$

*If parameters* $t_{1},t_{2}=0$, *the corresponding inequalities for the Zipf law follow*:

46 $$ \frac{1}{2N^{s_{2}}H_{N,s_{2}}} \tilde{\Lambda}^{2}_{h} \leq h^{2}(i, N, s_{1}, s_{2})\leq \frac{1}{2H_{N,s_{2}}} \tilde{\Lambda}^{2}_{h}, $$

*where*

$$\begin{aligned} \tilde{\Lambda}^{2}_{h}=\sum _{i=1}^{N} \biggl(1-\sqrt {i^{s_{1}-s_{2}} \frac{H_{N,s_{1}}}{H_{N,s_{2}}}} \biggr)^{2}-N \Biggl(1-\sqrt{ \frac{1}{N}\sum_{i=1}^{N} i^{s_{1}-s^{2}} \frac {H_{N,s_{1}}}{H_{N,s_{2}}}} \Biggr)^{2}. \end{aligned}$$

### Proof

Inequalities (45) follow from Corollary 8 as a special case of inequalities (37), for the convex function $t\mapsto\frac {1}{2} (\sqrt{t}-1 )^{2}$.

Similarly, inequalities (46) follow from Corollary 9 as a special case of inequalities (39). □

In the sequel we provide the results of this type for the Bhattacharyya coefficient (13), starting with one *N*-tuple defined via the Zipf–Mandelbrot law and proceeding with both such *N*-tuples, as well as with the Zipf law, as its special case.

### Corollary 14

*Let* $\mathbf{p}=(p_{1},\ldots,p_{N})$ *be an* *N*-*tuple of nonnegative real numbers with* $P_{N}=\sum_{i=1}^{N}p_{i}$, $N \in\mathbb{N}$ *and* $s_{2}> 0$, $t_{2}\geq0$.

*Then*

47 $$\begin{aligned} 1-\frac{1}{(N+t_{2})^{s_{2}}H_{N,s_{2},t_{2}}}\Lambda^{1}_{B} \geq B(i, N, s_{2},t_{2},\mathbf{p},) \geq1-\frac{1}{(1+t_{2})^{s_{2}}H_{N,s_{2},t_{2}}}\Lambda^{1}_{B}, \end{aligned}$$

*where*

$$\begin{aligned} \Lambda^{1}_{B}=\sqrt{N\sum _{i=1}^{N}p_{i}(i+t_{2})^{s_{2}}H_{N,s_{2},t_{2}}}- \sum_{i=1}^{N}\sqrt {p_{i}(i+t_{2})^{s_{2}}H_{N,s_{2},t_{2}}}. \end{aligned}$$

### Proof

It follows from Corollary 7 as a special case of inequalities (35), for the convex function $t\mapsto-\sqrt{t}$. It can also be derived from Corollary 3 and Remark 5 in the context of the Zipf–Mandelbrot law. □

### Corollary 15

*Let* $N \in\mathbb{N}$ *and* $s_{1}, s_{2}> 0$, $t_{1},t_{2}\geq0$.

*Then*

48 $$\begin{aligned} 1-\frac{1}{(N+t_{2})^{s_{2}}H_{N,s_{2},t_{2}}}\Lambda^{2}_{B} \geq B(i, N,s_{1}, t_{1},s_{2},t_{2}) \geq1-\frac{1}{(1+t_{2})^{s_{2}}H_{N,s_{2},t_{2}}}\Lambda^{2}_{B}, \end{aligned}$$

*where*

$$\begin{aligned} \Lambda^{2}_{B}=\sqrt{N\sum _{i=1}^{N}\frac {(i+t_{2})^{s_{2}}H_{N,s_{2},t_{2}}}{(i+t_{1})^{s_{1}}H_{N,s_{1},t_{1}}}}- \sum _{i=1}^{N}\sqrt{\frac {(i+t_{2})^{s_{2}}H_{N,s_{2},t_{2}}}{(i+t_{1})^{s_{1}}H_{N,s_{1},t_{1}}}}. \end{aligned}$$

*If parameters* $t_{1},t_{2}=0$, *the corresponding inequalities for the Zipf law follow*:

49 $$\begin{aligned} 1-\frac{1}{N^{s_{2}}H_{N,s_{2}}}\tilde{\Lambda}^{2}_{B} \geq B(i, N,s_{1}, s_{2}) \geq1- \frac{1}{H_{N,s_{2}}}\tilde{\Lambda}^{2}_{B}, \end{aligned}$$

*where*

$$\begin{aligned} \tilde{\Lambda}^{2}_{B}=\sqrt{N\sum _{i=1}^{N}i^{s_{2}-s_{1}}\frac {H_{N,s_{2}}}{H_{N,s_{1}}}}- \sum _{i=1}^{N}\sqrt{i^{s_{2}-s_{1}} \frac{H_{N,s_{2}}}{H_{N,s_{1}}}}. \end{aligned}$$

### Proof

Inequalities (48) follow from Corollary 8 as a special case of inequalities (37), for the convex function $t\mapsto-\sqrt{t}$.

Similarly, inequalities (49) follow from Corollary 9 as a special case of inequalities (39). □

In the same manner we proceed with analogous results for the chi-square divergence (14) and the total variation distance (15).

### Corollary 16

*Let* $\mathbf{p}=(p_{1},\ldots,p_{N})$ *be an* *N*-*tuple of real numbers with* $P_{N}=\sum_{i=1}^{N}p_{i}$, $N \in\mathbb{N}$ *and* $s_{2}> 0$, $t_{2}\geq0$.

*Then*

50 $$\begin{aligned} \frac{1}{(N+t_{2})^{s_{2}}H_{N,s_{2},t_{2}}}\Lambda^{1}_{\mathrm{chi}} \leq \chi^{2}(i, N, s_{2},t_{2},\mathbf{p}) \leq \frac{1}{(1+t_{2})^{s_{2}}H_{N,s_{2},t_{2}}}\Lambda^{1}_{\mathrm{chi}}, \end{aligned}$$

*where*

$$\begin{aligned} \Lambda^{1}_{\mathrm{chi}}=\sum_{i=1}^{N} \bigl(p_{i}(i+t_{2})^{s_{2}}H_{N,s_{2},t_{2}}-1 \bigr)^{2}-N \Biggl(\frac {1}{N}\sum_{i=1}^{N}p_{i}(i+t_{2})^{s_{2}}H_{N,s_{2},t_{2}}-1 \Biggr)^{2}. \end{aligned}$$

### Proof

It follows from Corollary 7 as a special case of inequalities (35), for the convex function $t\mapsto(t-1)^{2}$. It can also be derived from Corollary 4 and Remark 6 in the context of the Zipf–Mandelbrot law. □

### Corollary 17

*Let* $N \in\mathbb{N}$ *and* $s_{1}, s_{2}> 0$, $t_{1},t_{2}\geq0$.

*Then*

51 $$\begin{aligned} \frac{1}{(N+t_{2})^{s_{2}}H_{N,s_{2},t_{2}}}\Lambda^{2}_{\mathrm{chi}} \leq \chi^{2}(i, N, s_{1},t_{1},s_{2},t_{2}) \leq\frac{1}{(1+t_{2})^{s_{2}}H_{N,s_{2},t_{2}}}\Lambda^{2}_{\mathrm{chi}}, \end{aligned}$$

*where*

$$\begin{aligned} \Lambda^{2}_{\mathrm{chi}}=\sum_{i=1}^{N} \biggl(\frac {(i+t_{2})^{s_{2}}H_{N,s_{2},t_{2}}}{(i+t_{1})^{s_{1}}H_{N,s_{1},t_{1}}}-1 \biggr)^{2}- N \Biggl(\frac{1}{N} \sum_{i=1}^{N}\frac {(i+t_{2})^{s_{2}}H_{N,s_{2},t_{2}}}{(i+t_{1})^{s_{1}}H_{N,s_{1},t_{1}}}-1 \Biggr)^{2}. \end{aligned}$$

*If the parameters* $t_{1},t_{2}=0$, *the corresponding inequalities for the Zipf law follow*:

52 $$\begin{aligned} \frac{1}{N^{s_{2}}H_{N,s_{2}}}\tilde{\Lambda}^{2}_{\mathrm{chi}} \leq\chi^{2}(i, N, s_{1},s_{2}) \leq \frac{1}{H_{N,s_{2}}}\tilde{\Lambda}^{2}_{\mathrm{chi}}, \end{aligned}$$

*where*

$$\begin{aligned} \tilde{\Lambda}^{2}_{\mathrm{chi}}=\sum_{i=1}^{N} \biggl(i^{s_{2}-s_{1}}\frac {H_{N,s_{2}}}{H_{N,s_{1}}}-1 \biggr)^{2}- N \Biggl( \frac{1}{N}\sum_{i=1}^{N}i^{s_{2}-s_{1}} \frac {H_{N,s_{2}}}{H_{N,s_{1}}}-1 \Biggr)^{2}. \end{aligned}$$

### Proof

Inequalities (51) follow from Corollary 8 as a special case of inequalities (37), for the convex function $t\mapsto(t-1)^{2}$.

Similarly, inequalities (52) follow from Corollary 9 as a special case of inequalities (39). □

### Corollary 18

*Let* $\mathbf{p}=(p_{1},\ldots,p_{N})$ *be an* *N*-*tuple of real numbers with* $P_{N}=\sum_{i=1}^{N}p_{i}$, $N \in\mathbb{N}$ *and* $s_{2}> 0$, $t_{2}\geq0$.

*Then*

53 $$\begin{aligned} \frac{1}{(N+t_{2})^{s_{2}}H_{N,s_{2},t_{2}}}\Lambda^{1}_{V}\leq V(i, N, s_{2},t_{2}, \mathbf{p}) \leq\frac {1}{(1+t_{2})^{s_{2}}H_{N,s_{2},t_{2}}} \Lambda^{1}_{V}, \end{aligned}$$

*where*

$$\begin{aligned} \Lambda^{1}_{V}=\sum_{i=1}^{N} \bigl\vert p_{i}(i+t_{2})^{s_{2}}H_{N,s_{2},t_{2}}-1 \bigr\vert -N \Biggl\vert \frac {1}{N}\sum_{i=1}^{N}p_{i}(i+t_{2})^{s_{2}}H_{N,s_{2},t_{2}}-1 \Biggr\vert . \end{aligned}$$

### Proof

It follows from Corollary 7 as a special case of inequalities (35), for the convex function $t\mapsto \vert t-1 \vert $. It can also be derived from Corollary 5 and Remark 7 in the context of the Zipf–Mandelbrot law. □

### Corollary 19

*Let* $N \in\mathbb{N}$ *and* $s_{1}, s_{2}> 0$, $t_{1},t_{2}\geq0$.

*Then*

54 $$\begin{aligned} \frac{1}{(N+t_{2})^{s_{2}}H_{N,s_{2},t_{2}}}\Lambda^{2}_{V}\leq V(i, N, s_{1},t_{1},s_{2},t_{2}) \leq \frac {1}{(1+t_{2})^{s_{2}}H_{N,s_{2},t_{2}}}\Lambda^{2}_{V}, \end{aligned}$$

*where*

$$\begin{aligned} \Lambda^{2}_{V}=\sum_{i=1}^{N} \biggl\vert \frac {(i+t_{2})^{s_{2}}H_{N,s_{2},t_{2}}}{(i+t_{1})^{s_{1}}H_{N,s_{1},t_{1}}}-1 \biggr\vert - N \Biggl\vert \frac{1}{N}\sum_{i=1}^{N} \frac {(i+t_{2})^{s_{2}}H_{N,s_{2},t_{2}}}{(i+t_{1})^{s_{1}}H_{N,s_{1},t_{1}}}-1 \Biggr\vert . \end{aligned}$$

*If parameters* $t_{1},t_{2}=0$, *the corresponding inequalities for the Zipf law follow*:

55 $$\begin{aligned} \frac{1}{N^{s_{2}}H_{N,s_{2}}}\tilde{\Lambda}^{2}_{V}\leq V(i, N, s_{1},s_{2}) \leq\frac{1}{H_{N,s_{2}}}\tilde{ \Lambda}^{2}_{V}, \end{aligned}$$

*where*

$$\begin{aligned} \tilde{\Lambda}^{2}_{V}=\sum_{i=1}^{N} \biggl\vert i^{s_{2}-s_{1}}\frac {H_{N,s_{2}}}{H_{N,s_{1}}}-1 \biggr\vert - N \Biggl\vert \frac{1}{N}\sum_{i=1}^{N}i^{s_{2}-s_{1}} \frac {H_{N,s_{2}}}{H_{N,s_{1}}}-1 \Biggr\vert . \end{aligned}$$

### Proof

Inequalities (54) follow from Corollary 8 as a special case of inequalities (37), for the convex function $t\mapsto \vert t-1 \vert $.

Similarly, inequalities (55) follow from Corollary 9 as a special case of inequalities (39). □

In order to conclude this section providing the Jensen-inequality related results for the *f*-divergences based on the Zipf–Mandelbrot law (or the Zipf law), for the triangular discrimination (16) we give only the latter one: the bounds obtained in the case of both *N*-tuples observed via the Zipf law.

### Corollary 20

*Let* $N \in\mathbb{N}$ *and* $s_{1}, s_{2}> 0$.

*Then*

56 $$\begin{aligned} \frac{1}{N^{s_{2}}H_{N,s_{2}}}\tilde{\Lambda}^{2}_{\Delta} \leq \Delta(i, N, s_{1},s_{2}) \leq\frac{1}{H_{N,s_{2}}}\tilde{ \Lambda }^{2}_{\Delta}, \end{aligned}$$

*where*

$$\begin{aligned} \tilde{\Lambda}^{2}_{\Delta}=\sum_{i=1}^{N} \frac{ (i^{s_{2}}H_{N,s_{2}}- i^{s_{1}}H_{N,s_{1}} )^{2}}{i^{s_{1}}H_{N,s_{1}} (i^{s_{1}}H_{N,s_{1}}+i^{s_{2}}H_{N,s_{2}} )}- N\frac{ (\frac{1}{N}\sum_{i=1}^{N}i^{s_{2}-s_{1}}\frac {H_{N,s_{2}}}{H_{N,s_{1}}} -1 )^{2}}{\frac{1}{N}\sum_{i=1}^{N}i^{s_{2}-s_{1}}\frac{H_{N,s_{2}}}{H_{N,s_{1}}}+1}. \end{aligned}$$

### Proof

The inequalities can easily be deduced from Corollary 9 as a special case of inequalities (39), for the convex function $t\mapsto\frac {(t-1)^{2}}{t+1}$. It can also be derived from Corollary 6 and Remark 8 in the context of the Zipf law. □

## An application of the Zipf law

In the final section we are going to show how the experimental character of the Zipf law can be interpreted through the bounds (43) obtained for the Kullback–Leibler divergence.

Namely, the coefficients $s_{1}$ and $s_{2}$ from the Zipf law were analyzed by Gelbukh and Sidorov in [25] as assigned to the Russian and English languages. They calculated the mentioned coefficients and their difference for each of the 39 literature texts in both languages, with more than 10,000 running words inside of each of them. In the process they obtained the average of $s_{1}=0.892869$ for the Russian and $s_{2}=0.973863$ for the English language.

In this context, with the described experimental values of $s_{1}$ and $s_{2}$ involved, the bounds for the Kullback–Leibler divergence in (43) assume the following form which thus depends only on the parameter *N*.

### Example 1

Let $\mathbf{p}=(p_{1},\ldots,p_{N})$ and $\mathbf{q}=(q_{1},\ldots ,q_{N})$ be distributions associated to the Russian and English languages, respectively, and let $N\in\mathbb{N}$ be a parameter. If the logarithm base is greater than 1, then

57 $$ \frac{1}{N^{0.973863}H_{N,0.973863}} \tilde{\Lambda}^{2}_{\mathrm{KL}} \leq \mathrm{KL}(i,N)\leq\frac{1}{H_{N,0.973863}} \tilde{\Lambda}^{2}_{\mathrm{KL}}, $$

where

$$\begin{aligned} \tilde{\Lambda}^{2}_{\mathrm{KL}}={}&\sum _{i=1}^{N}\frac{i^{0.080994} H_{N,0.973863}}{H_{N,0.892869}} \log \biggl( \frac{i^{0.080994} H_{N,0.973863}}{H_{N,0.892869}} \biggr) \\ & {}-\sum_{i=1}^{N} \frac{i^{0.080994} H_{N,0.973863}}{H_{N,0.892869}} \log \Biggl(\frac{1}{N}\sum _{1=1}^{N}\frac{i^{0.080994} H_{N,0.973863}}{H_{N,0.892869}} \Biggr). \end{aligned}$$

### Proof

It follows directly from (43) when inserting the experimental values of $s_{1}$ and $s_{2}$. □

## Conclusions

In this paper we investigated *f*-divergences that originate from the Csiszár functional and their link to the Jensen inequality with a specific type of the Jensen-type interpolating inequalities. By means of these inequalities we derived new bounds for *f*-divergences in general via the Csiszár functional and in particular for the Kullback–Leibler divergence, Hellinger distance, Bhattacharyya distance (coefficient), $\chi^{2}$-divergence, total variation distance and triangular discrimination. Consequently, we deduced analogous results in the light of the well-known Zipf–Mandelbrot law, with the adequate probability mass functions and the adjusted form of the Csiszár functional. The Zipf–Mandelbrot law was analyzed as a more general form of the Zipf law, for which we also gave the corresponding results and an application in linguistics in order to accentuate its experimental character. Thus this paper includes three important and widely investigated issues: the Jensen inequality, the divergences (for probability distributions) and the Zipf–Mandelbrot law with its less general, but not less important form, the Zipf law. In this way, the paper can be of an interest for mathematicians who investigate any of these fields with an accent put on mathematical inequalities, as well as for the interdisciplinary fields (*e.g.* linguistics was involved in this case).
